# Effects of Sex, Smoking, and Physical Activity on Metabolic Syndrome Among Current Smokers: A Cross-Sectional Study from Taiwan

**DOI:** 10.3390/healthcare13212678

**Published:** 2025-10-23

**Authors:** Ke-Ting Pan, Fan-Min Lin, Ta-Wei Chu, Ming-Tsung Chen, Yuan-Chieh Chuang, Dee Pei, Chih-Hao Shen

**Affiliations:** 1 Graduate Institute of Aerospace and Undersea Medicine, College of Biomedical Sciences, National Defense Medical University, Taipei 11490, Taiwan; pankt930@gmail.com; 2 Center for Gender and Health Studies, National Defense Medical University, Taipei 11490, Taiwan; 3 Division of Pulmonary Medicine, Department of Internal Medicine, Kaohsiung Armed Forces General Hospital, Kaohsiung 80284, Taiwan; a0968020002@mail.802.org.tw; 4 MJ Health Research Foundation, Taipei 114066, Taiwan; 5 Department of Obstetrics and Gynecology, Tri-Service General Hospital, National Defense Medical University, Taipei 114202, Taiwan; 6 Division of Pulmonary and Critical Care Medicine, Department of Internal Medicine, Tri-Service General Hospital, National Defense Medical University, Taipei 114202, Taiwan; 7 Department of Medicine, Medical School, Fu Jen Catholic University, New Taipei City 242062, Taiwan; 8 Department of Endocrinology and Metabolism, Fu Jen Catholic University Hospital, New Taipei City 242062, Taiwan

**Keywords:** gender differences, tobacco use, exercise, lifestyle behaviors, Taiwan population

## Abstract

**Background**: Metabolic syndrome (MetS) is a growing global health concern. Although sex, smoking, and physical activity are recognized risk factors, their combined effects remain insufficiently studied, particularly among Asian populations. This study aimed to examine the associations of sex, cumulative smoking exposure, and physical activity with MetS among current smokers in Taiwan. **Methods**: Data were drawn from 15,385 participants recruited between 2013 and 2017 from a health screening center. Demographic characteristics, smoking status, physical activity levels, and biochemical data were analyzed. Mann–Whitney U tests, chi-square tests, and multiple logistic regression were used to identify variables associated with MetS. **Results**: MetS prevalence differed significantly by sex, with rates of 13.6% in men and 5.1% in women. Women had a lower chance of developing MetS compared to men (OR = 0.607, 95% CI 0.488–0.754). Older age, higher body mass index, and greater cumulative smoking exposure (quantified using a composite ‘smoke area’ index derived from questionnaire data on smoking duration, frequency, and daily amount) were positively associated with MetS risk. Among smokers younger than 45 years, women also had significantly lower odds of MetS than men (OR = 0.590, 95% CI 0.451–0.771). Higher levels of physical activity were linked to reduced MetS risk in both sexes. **Conclusions**: Among current smokers, being female was inversely associated with the risk of MetS. Greater physical activity and lower smoking exposure were also associated with reduced risk. Future research should use longitudinal designs and comorbidities to clarify mechanisms and inform tailored prevention strategies.

## 1. Introduction

Metabolic syndrome (MetS) refers to a cluster of five health conditions, including abdominal obesity, high blood pressure, impaired fasting plasma glucose (FPG), high triglyceride levels, and low high-density lipoprotein cholesterol (HDL-C). Metabolic syndrome is diagnosed in patients who have three or more of the five health conditions [1]. Metabolic syndrome raises a patient’s risk for chronic diseases, such as cancer, stroke, and diabetes, as well as other serious health problems, and its pathophysiology involves several complex mechanisms, such as insulin resistance, chronic inflammation, and neurohormonal activation [2,3]. The global prevalence of MetS has been rising in parallel with aging populations, sedentary lifestyles, and unhealthy diets, making it a critical public health challenge worldwide [3,4].

In previous studies, cigarette smoking has also been shown to increase one’s risk of developing MetS [5,6,7,8]. Cigarette smoke consists of over 4000 chemical compounds, including toxins and electrophiles; these compounds cause oxidative stress, which triggers chronic inflammation [9]. In a cross-sectional survey conducted in China with 15,222 participants aged 60 and over, cigarette smoking throughout life was associated with increased risks for MetS [6]. Another Korean study involving individuals under the age of 40 found that the risk of MetS was significantly higher among smokers than nonsmokers [7]. A retrospective study of 22,892 Japanese people revealed that both current and past smoking habits contributed to the occurrence of MetS [8]. However, the relationship is not entirely consistent. For example, a Turkish cohort study unexpectedly suggested a “protective” effect of heavy smoking against MetS in women [10]. This counterintuitive finding may reflect cultural factors influencing smoking behavior and underscores the need for critical evaluation and replication in other populations.

There are also sex disparities in cigarette smoking-related cardiovascular and metabolic disease risks [11]. Compared to nonsmokers, current male smokers exhibited higher circulating C-reactive protein (CRP) levels, whereas current female smokers had similar CRP levels [12]. Since CRP is strongly linked to cardiovascular outcomes, these differences may contribute to sex-specific variations in metabolic risk. In a study on American Indians with diabetes, cardiovascular disease in current female smokers had a hazard ratio of 1.00 compared to nonsmokers, whereas the hazard ratio in male smokers was 1.49 [13]. Such findings raise questions about whether hormonal or fat-distribution differences might mitigate certain risks in women, despite smoking’s overall harmful effects.

Studies have also shown that physical activity is negatively correlated with MetS risk [14,15,16,17,18,19,20]. Increased physical activity may help to reduce MetS risk through its effects on insulin resistance, adipose fuel metabolism, inflammation, and epigenetic factors [14]. Sex also affects the correlation between physical activity and MetS. A study using National Health and Nutrition Examination Survey data found that physical activity can lower MetS risk among young and middle-aged men and women. Longer durations of physical activity seemed to lower the risk of developing MetS more in men than in women; however, this sex difference lessened with age [15]. These observations highlight the importance of considering sex and activity interactions when studying MetS.

Despite these insights, important gaps remain. In Taiwan, male smoking prevalence has historically been high but has shown a gradual decline in recent years, whereas female smoking rates, though still lower, have been steadily increasing [16]. Yet, little is known about how sex differences influence the relationship between smoking and MetS in this context. Moreover, most studies classify smoking status simply as ‘current’, ‘former’, or ‘never’, without quantifying cumulative exposure, making it difficult to capture the full impact of long-term smoking behavior [6,7]. Finally, although physical activity is recognized as protective, few studies have examined how it may modify the sex-specific effects of smoking on MetS, particularly in Asian populations.

Therefore, the present study aims to (1) evaluate the effects of sex, cumulative smoking exposure, and physical activity on the development of MetS among current smokers in Taiwan and (2) examine laboratory indicators of lipid metabolism, including serum triglycerides, HDL-C, and LDL-C, as part of the assessment of metabolic status. By combining behavioral and biological measures, this study seeks to provide a more comprehensive understanding of the determinants of MetS in current smokers among Taiwan’s population.

## 2. Methods

**Figure 1:**
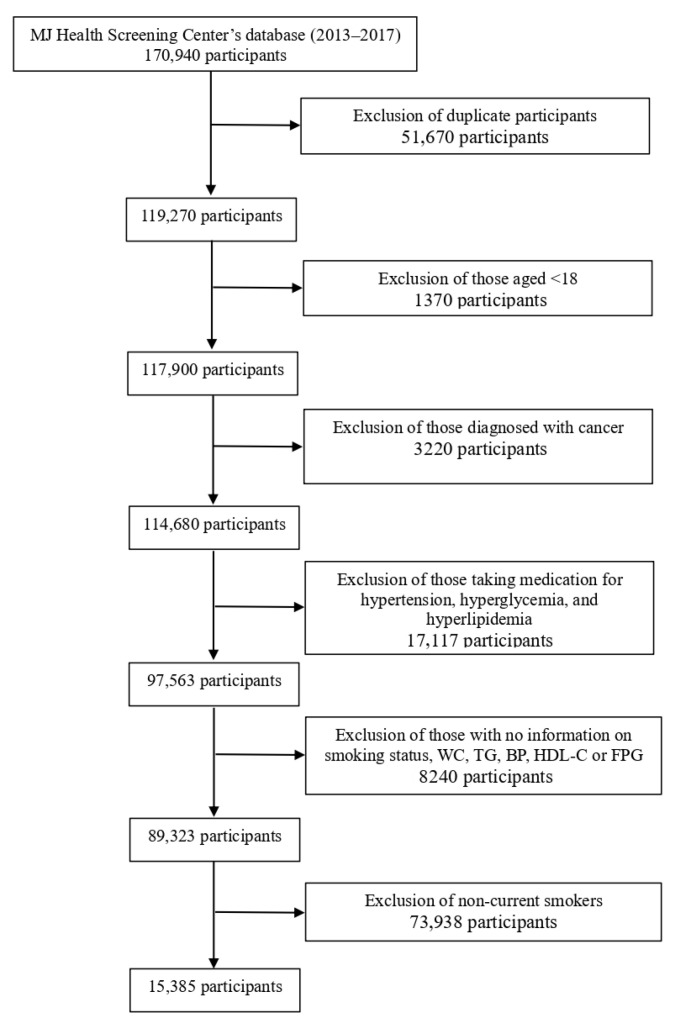
Flowchart of the participant selection process.

This study involved screening 170,940 patient records created between 1 January 2013 and 31 December 2017 These records come from the MJ Health Screening Center’s database, a large database and biorepository of data from healthy individuals gathered as part of a health screening program conducted by the largest health management institute in Taiwan. Participant data were entered anonymously during routine health examinations and used for research purposes only. In the event of multiple records existing for a single participant, only their first record was used. Moreover, records were excluded for participants under the age of 18; those diagnosed with cancer, to avoid complex physiological effects that differ from the general population; those taking medication for hypertension, hyperglycemia, or hyperlipidemia, to minimize the potential confounding effects of pharmacological treatment on metabolic parameters; those with missing data; and those who were not current smokers. Resultantly, 15,385 eligible participants were recruited for the study (Figure 1).

This study was approved by the Institutional Review Board of the Tri-Service General Hospital on 2 May 2022 (TSGHIRB Approval No. B202205087). We used secondary health data from the MJ Health Screening Center database. At the time of health examinations, the center obtained written informed consent from all participants for their data to be used in research. For the present analysis, we accessed only anonymized data provided by the MJ Health Research Foundation; therefore, the requirement for additional informed consent was waived by the Institutional Review Board.

### 2.1. Anthropometric Measurements and General Data

Senior nursing staff members used a questionnaire to obtain participants’ medical histories, including currently prescribed medications. Complete physical examinations were also performed. Body mass index (BMI) was calculated by dividing a participant’s body weight (kg) by the square of the participant’s height (m). Systolic blood pressure (SBP) and diastolic blood pressure (DBP) were measured by nursing staff using standard mercury sphygmomanometers fitted on the right arm of each participant while seated. Smoking and physical activity data were obtained from self-reported questionnaires administered during health examinations. For current smokers, cumulative smoking exposure was defined as the frequency of smoking multiplied by the smoking amount and then the years of smoking. Three items were included: (1) smoking duration (years), (2) smoking frequency (never, former, occasional, daily), and (3) smoking amount (average daily cigarette consumption). Each item was converted into a numeric score, and the three scores were multiplied to generate a composite indicator (‘smoke area’) representing cumulative smoking exposure (Supplementary Table S1). This index is unitless but reflects the combined contribution of smoking duration, frequency, and intensity. The metabolic equivalent of task (MET) was defined as follows: 1 MET = 1 kcal per hour per kg of body weight. An MET of 2.5 is defined as a light exercise, while an MET of 4.5 is moderate, 6.5 is medium–vigorous, and 8.5 is high–vigorous exercise [21,22]. Moreover, MET-hr was defined as MET multiplied by the number of hours of exercise.

In this study, an MetS diagnosis required that the patient possess three of the five health conditions [23], including increased waist circumference (WC) (men ≥ 102 cm, women ≥ 88 cm), high triglyceride (TG) levels (≥150 milligrams per deciliter of blood, mg/dL), reduced HDL-C (<40 mg/dL in men; <50 mg/dL in women), elevated FPG (≥100 mg/dL), and high blood pressure (SBP ≥ 130 mmHg; DBP ≥ 85 mmHg).

Laboratory measurements were conducted after the participant fasted for 10 h. Blood samples were drawn from the antecubital vein for biochemical analysis. Plasma was separated from the blood within 1 h, stored at −30 °C in laboratory refrigerators, and analyzed for FPG and lipid profiles. The FPG was measured using the glucose oxidase method with a YSI 203 glucose analyzer (Yellow Springs Instruments, Yellow Springs, OH, USA). Total cholesterol (TC) and TG were measured using the dry, multilayer analytical slide method with the Fuji Dri-Chem 3000 analyzer (Fuji Photo Film, Minato-Ku, Tokyo, Japan). Serum HDL-C and low-density lipoprotein cholesterol (LDL-C) concentrations were analyzed using the enzymatic cholesterol assay method after performing dextran sulfate precipitation. Hemoglobin was measured with an Abbott Cell Dyn 3000 hematology analyzer (Abbott Laboratories, Abbott Park, IL, USA). To ensure methodological rigor, instruments were calibrated regularly according to manufacturer instructions, and internal quality control procedures were performed daily.

### 2.2. Statistical Analysis

All statistical analyses were performed using IBM SPSS Statistics version 29.0 (IBM Corp., Armonk, NY, USA). Data are presented as mean ± standard deviation. All data were tested for normal distribution using the Kolmogorov–Smirnov test. The Mann–Whitney U and chi-square tests were applied to compare differences between variables and MetS distribution among men and women. The relationship between cumulative smoking exposure and continuous variables was analyzed via a correlation test. Subsequently, multiple logistic regression was conducted to examine the associations of sex, cumulative smoking exposure, and physical activity with MetS, while adjusting for age and BMI.

## 3. Results

Table 1 compares the demographic characteristics, biochemistry, and MetS distribution of men and women, revealing significant differences (*p* < 0.001) according to sex for all parameters. The sample included 12,541 current male smokers and 2844 current female smokers. The cumulative smoking exposure was 33.1 pack-years in current male smokers and 22.1 pack-years in current female smokers (*p* < 0.001). The distribution of MetS between men and women differed significantly: Incidence rates of MetS for men and women were 13.6% and 5.1%, respectively (*p* < 0.001). Other parameters, including age, height, weight, BMI, WC, MET-hr, FPG, blood pressure, cholesterol, and TG also differed significantly between women and men (*p* < 0.001).

To validate the effects of sex hormones on MetS, we evaluated current smokers under the age of 45 while ensuring to include only women who had not yet experienced menopause. Table 2 also illustrates differences between men and women under the age of 45 who are current smokers in terms of demographic characteristics, biochemistry, and MetS distribution. All parameters differed significantly between men and women. Mean age, height, weight, BMI, WC, MET-hr, cumulative smoking exposure, FPG, BP, TC, LDL-C, TG, and MetS incidence were higher in men than in women (*p* < 0.001). Men, however, had lower HDL-C levels than women (*p* < 0.001).

Table 3 illustrates the correlations between cumulative smoking exposure and various parameters (*, *p* < 0.05). For men, cumulative smoking exposure was positively correlated with age, BMI, WC, FPG, DBP, TC, LDL-C, and TG and negatively correlated with MET-hr and HDL-C. For women, cumulative smoking exposure was positively correlated with age, WC, FPG, TC, LDL-C, and TG and negatively correlated with MET-hr and HDL-C. Among current smokers under the age of 45, for men, cumulative smoking exposure was positively correlated with age, BMI, WC, FPG, DBP, TC, LDL-C, and TG and was negatively correlated with MET-hr and HDL-C. For women, cumulative smoking exposure was positively correlated with age, TC, LDL-C, and TG and was negatively correlated with MET-hr and HDL-C.

Table 4 demonstrates that age, sex, BMI, cumulative smoking exposure, and MET-hr may be significant parameters for predicting MetS. Women may have a lower chance of developing MetS than men (odds ratio [OR] = 0.524, 95% CI 0.470–0.583). The OR was 0.995 (95% CI 0.992–0.998) for MET-hr, indicating that participants with a higher MET-hr may have a lower chance of developing MetS. However, the OR was 1.051 (95% CI 1.046–1.056) for age, 1.264 (95% CI 1.247–1.281) for BMI, and 1.004 (95% CI 1.002–1.006) for cumulative smoking exposure, demonstrating that older participants with higher BMI and cumulative smoking exposure are at higher risk for devel-oping MetS. Among current smokers under the age of 45, women may have a lower chance of developing MetS than men (OR = 0.505, 95% CI 0.448–0.569). The OR was 1.062 (95% CI 1.053–1.071) for age, 1.257 (95% CI 1.239–1.276) for BMI, and 1.004 (95% CI 1.001–1.006) for cumulative smoking exposure, indicating that older participants with higher BMI and cumulative smoking exposure are at greater risk for developing MetS. However, MET-hr was also a significant protective predictor of MetS development among current smokers under the age of 45 (OR = 0.995, 95% CI 0.992–0.998).

**Figure 2:**
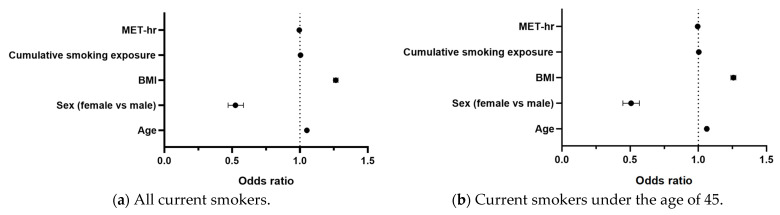
Forest plot of logistic regression analysis of factors associated with MetS. Forest plot of odds ratios (ORs) with 95% confidence intervals for factors associated with MetS among current smokers. Variables included in the model were age, sex, BMI, cumulative smoking exposure, and MET-hr. The vertical dashed line indicates the null value (OR = 1).

The results of the multiple logistic regression are presented in Table 4 and summarized graphically in Figure 2. The forest plot illustrates that older age, higher BMI, and greater cumulative smoking exposure were positively associated with MetS, whereas female sex and higher physical activity were inversely associated with MetS.

A detailed breakdown of the five components of MetS is presented in Tables S1–S5. Among current smokers, female sex appeared to be a protective factor against elevated TG, BP, and FPG, with ORs of 0.377, 0.266, and 0.489, respectively. Conversely, female sex was identified as a risk factor for increased WC and HDL-C, with ORs of 10.402 and 2.137, respectively. Cumulative smoking exposure was significantly associated with elevated TG and low HDL-C levels (OR = 1.013 and 1.008). In contrast, MET-hr, was a significant protective factor against elevated WC, TG, FPG, and low HDL-C, with ORs of 0.990, 0.993, 0.996, and 0.993, respectively.

## 4. Discussion

This study aimed to elucidate the effects of sex, cumulative smoking, and physical activity on MetS development among current smokers in Taiwan. In this study, being female and having higher levels of physical activity (MET-hr) were shown to be protective against MetS, while elevated cumulative smoking exposure was revealed as a risk factor for MetS among current smokers. Although the effects of cumulative smoking exposure and physical activity seemed to differ between men and women, the participant’s sex itself was shown to have a greater impact on MetS among current smokers. Cumulative smoking exposure was positively correlated with higher age, WC, FPG, TG, TC, and LDL-C and was negatively correlated with HDL-C and MET-hr among all current smokers. Moreover, although cumulative smoking exposure was positively correlated with BMI among current male smokers, no relationship was found among current female smokers. To our knowledge, this is the first study to examine the effects of sex and physical activity on MetS among current smokers in Taiwan.

This study found that being female was a protective factor against MetS development in current smokers independent of age, BMI, cumulative smoking exposure, and physical activity level. Previous studies have shown that the mechanisms underlying metabolic-related diseases have sex-specific components and that inflammatory processes have been implicated in the pathogenesis of MetS [24,25]. In both animal and human studies, sex hormones, particularly estrogen, have been shown to regulate energy metabolism, food intake, and body weight [25]. Estrogen can increase fat oxidation, inhibit lipogenesis, and improve adipogenic potential in gluteofemoral adipocytes, thus attenuating systemic inflammation [26,27]. In animal models of diet-induced obesity, female rats experience less risk of hyperglycemia, hyperinsulinemia, and hypertension than their male counterparts (Supplementary Tables S5 and S6) [28,29]. Smoking is a well-established factor for MetS that triggers the release of inflammatory mediators, including lipoprotein lipase, adiponectin, peroxisome proliferator-activated receptors, and tumor necrosis factor-alpha [1], and on this basis, it can be hypothesized that sex hormones may also reduce the risk of smoking-related MetS by alleviating chronic inflammation in women.

Previous studies have shown that menopause increases women’s risk for developing MetS and associated diseases, such as obesity, cardiovascular diseases, and type 2 diabetes mellitus [30]. These risks can be reduced via hormone replacement therapy, demonstrating the importance of estrogen signaling in these processes [31]. Estrogen levels and histories of hormone replacement therapy were not recorded in the database used for this study; therefore, to validate the effects of sex hormones on MetS, we limited the study sample to current smokers under the age of 45 while also ensuring that only women who had not yet experienced menopause were included. We found that women in this subgroup displayed a lower predicted change in odds (Exp [B]) (Table 4) for MetS compared to the overall database population, highlighting the protective role of sex hormones for smoking in women.

Major increases in the incidence of MetS and related diseases have been observed worldwide among people with less active lifestyles and increased food consumption [32]. Many studies have revealed that physical activity plays a role in reducing the risk for MetS [14,15,18,19]. In addition, engaging in moderate- to high-intensity physical activity can attenuate the risk of smoking-related MetS [33]. In this study, the intensity of physical activity was evaluated using MET-hr among current smokers. We found that MET-hr was an independent predictor for MetS. In a clinical trial, aerobic exercise training improved some MetS-associated pathophysiological changes, such as reduced arterial stiffening [34]. In our study, multiple regression showed that MET-hr was a protective factor of MetS for current smokers of all ages and under the age of 45 (Table 4).

A community cohort study in Taiwan found that sex is a critical factor affecting the relationship between physical activity and risk of MetS. The relationship between the frequency of physical activity and MetS was linear in women and exponential in men [17]. Body composition and physical activity preferences were also found to differ between men and women. In general, men displayed higher energy expenditure per minute than women. Therefore, among men and women who engage in similar levels of physical activity, men experienced greater benefits from physical activity than women [35]. Moreover, men are more likely to prefer outdoor activities than women, which may allow men to achieve higher vitamin D concentrations and reduce their SBP more than women [17]. However, a HERITAGE family study showed that exercise training can improve TG, HDL-C, TC, BP, FPG, and WC and enhance the efficacy of exercise equally among both men and women [36]. Meanwhile, our study showed that physical activity can reduce the risk of MetS in both men and women.

Smoking has been well-established as a contributing factor for MetS [7,33]. In this study, cumulative smoking exposure was revealed as an important factor that increased MetS risk in current smokers. For both men and women, MET-hr decreased significantly, whereas cumulative smoking exposure increased (Table 3). In previous studies, a similar trend was found—that is, smokers tended to have lower physical activity levels [37,38]. Moreover, smoking may adversely affect lung function [39]. Therefore, smokers may feel more tired, leading to diminished physical activity. However, the negative correlation between MET-hr and cumulative smoking exposure was less pronounced in women than in men. Previous findings regarding the relationship between smoking and physical activity for both men and women have been controversial [40,41]. Various behavioral, psychological, and sociocultural environmental factors may motivate female smokers to maintain their physical activity levels, and therefore, further studies are warranted.

Both previous studies and the present study have identified higher BMI and smoking as risk factors for MetS (Table 4) [42,43]. However, other studies have shown that current smokers have lower BMI than nonsmokers [37,44,45]. The mechanism through which smoking decreases BMI may be mediated by nicotine, which acts on nicotinic cholinergic receptors in the brain and autonomic ganglia [46]. Smoking may also serve as a behavioral alternative to eating, resulting in decreased food consumption [47]. A longitudinal study found that smoking over 20 cigarettes per day may result in weight gain regardless of sex [48]. This study found that BMI is positively correlated with cumulative smoking exposure in men but not in women (Table 3). Therefore, although women are better protected from the sequelae of obesity than men, it remains undetermined whether sex differences influence the occurrence of obesity [25].

A seemingly paradoxical finding of this study was that female smokers exhibited higher WC yet a lower prevalence of MetS compared with male smokers. Smoking affects parameters that determine obesity, including BMI and WC. Visceral obesity appears to be a major component of MetS, and abdominal obesity measured by WC—which is associated with visceral obesity—is considered to be more strongly correlated with the cardiovascular risks of obesity [49]. Sex-specific differences in fat distribution may partly explain this finding: women predominantly accumulate subcutaneous rather than visceral adipose tissue, whereas men are more prone to visceral fat deposition, which is metabolically more detrimental [50,51], a pattern also observed in our data (Supplementary Table S2). Furthermore, estrogen exerts favorable effects on lipid metabolism, including increasing HDL-C levels, which may partially offset the adverse metabolic consequences of central adiposity [52,53]. Therefore, these physiological and hormonal mechanisms may help explain the inverse association observed between female sex and MetS despite greater WC values.

Despite an overall reduction in BMI, smokers have a higher prevalence of central obesity compared to nonsmokers [1]. In this study, BMI was not correlated with cumulative smoking among women; however, a positive correlation was found between cumulative smoking and WC for both men and women. Although the correlation between cumulative smoking and WC was found to be lower in women than in men, the risks of obesity-related complications still require vigilance and must be prevented.

A meta-analysis showed that compared to nonsmokers, smokers had higher serum concentrations of TGs (9.1%) and LDL (1.7%) and lower HDL (−5.7%), as demonstrated in Supplementary Tables S3 and S4. Moreover, a dose-dependent relationship was shown for each variable when comparing nonsmokers with light, moderate, and heavy smokers [54]. Clinical studies also verify that smoking is associated with the risks of hyperglycemia, hyperinsulinemia, insulin resistance, and diabetes [54,55]. An acute increase in BP after smoking has been noted in previous studies; however, the chronic effects of this remain uncertain [1]. Whether sex differences influence lipid profile, FPG, and blood pressure in current smokers remains undetermined. In this study, cumulative smoking was positively correlated with increased TG, TC, LDL, and FPG and was negatively correlated with HDL-C in both sexes. Cumulative smoking was also positively correlated with DBP in men but not in women. In a previous study, men with hypertension were more likely to be smokers than men with normal BP. The prevalence of smoking was not significantly different between women with and without hypertension, however [56]. In addition, being female alleviated the changes in these parameters caused by smoking in a subgroup of people under the age of 45. These results may provide evidence supporting women’s advantages in smoking-related MetS.

This study has some limitations. First, the cross-sectional design used for this study is only able to capture correlations between factors; it cannot establish causal relationships between factors. Second, although cumulative smoking exposure and MET-hr were used to assess smoking levels and physical activity, these data were self-reported, which may introduce recall bias and reduce measurement accuracy. Third, as the data were obtained from the MJ Health Screening Center’s database, certain important variables were unavailable. These included hormone therapy and menopausal status, which limited our ability to assess the effects of female sex hormones and their interaction with smoking exposure and physical activity, as well as other clinical, environmental, and lifestyle factors such as anemia or COPD status, cadmium levels, diet, alcohol intake, and socioeconomic indicators. The absence of these data may have influenced the observed associations with MetS. Fourth, participants taking antihypertensive, antidiabetic, or lipid-lowering medications were excluded. While this approach minimized the potential confounding effects of pharmacological treatment on metabolic parameters, it also reduces the generalizability of our findings to the broader smoker population who may be receiving such therapies. Fifth, the relatively few women over 45 years may have constrained statistical power and influenced subgroup comparisons, warranting cautious interpretation of these findings. Sixth, cumulative smoking exposure was estimated using a composite ‘smoke area’ index rather than a standard measure such as pack-years; therefore, caution is needed when comparing our results with studies that used conventional smoking exposure metrics. Finally, as the MJ Health Screening Center includes individuals who voluntarily undergo routine health examinations, this population may be healthier, wealthier, or more health-conscious than the general Taiwanese smoking population. Thus, sampling bias cannot be excluded, and the generalizability of our findings should be interpreted with caution.

## 5. Conclusions

Among current smokers, being female was inversely associated with the risk of MetS. Furthermore, higher levels of physical activity and lower cumulative smoking exposure were associated with a reduced risk of MetS, regardless of sex. However, a closer examination of the five individual components of MetS suggests that sex, cumulative smoking exposure, and physical activity may exert differential associations with each component. These findings highlight the need for component-specific prevention strategies in addressing MetS. Future research should examine longitudinal trajectories of MetS development, incorporate additional lifestyle and environmental factors such as diet, cadmium exposure, and comorbidities, and evaluate potential interventions tailored to high-risk subgroups. These directions would provide more comprehensive insights into the mechanisms linking smoking and lifestyle behaviors with MetS.

## Figures and Tables

**Table 1:** Comparison of demographic characteristics, biochemistry, and MetS between current male and female smokers (all ages).

	All Current Smokers		
	Men *n* = 12,541 Mean ± SD	Women *n* = 2844 Mean ± SD	*p* Value
Age (year)	39.5 ± 10.0	36.6 ± 8.8	<0.001
Height (cm)	171.8 ± 6.2	159.8 ± 5.6	<0.001
Weight (kg)	73.1 ± 12.4	55.2 ± 10.2	<0.001
BMI (kg/m^2^)	24.7 ± 3.7	21.6 ± 3.8	<0.001
WC (cm)	84.3 ± 9.3	70.6 ± 8.6	<0.001
MET-hr (kcal/kg)	7.2 ± 15.2	4.4 ± 10.6	<0.001
Cumulative smoking exposure	33.1 ± 20.2	22.1 ± 18.3	<0.001
FPG (mmol/L)	102.6 ± 18.6	96.0 ± 11.9	<0.001
SBP (mmHg)	118.3 ± 14.8	103.9 ± 13.1	<0.001
DBP (mmHg)	75.7 ± 10.6	67.3 ± 9.4	<0.001
TC (mg/dL)	199.6 ± 37.1	189.7 ± 33.9	<0.001
HDL-C (mg/dL)	51.4 ± 11.4	65.5 ± 15.5	<0.001
LDL-C (mg/dL)	126.0 ± 34.6	108.4 ± 31.9	<0.001
TG (mg/dL)	153.0 ± 131.7	90.6 ± 65.9	<0.001
MetS			<0.001
Yes	1701 (13.6)	144 (5.1)	
No	10,840 (86.4)	2700 (94.9)	

**Table 2:** Comparison of demographic characteristics, biochemistry, and MetS between current male and female smokers younger than 45 years.

	Current Smokers Under the Age of 45		
	Men *n* = 9004 Mean ± SD	Women *n* = 2354 Mean ± SD	*p* Value
Age (year)	34.6 ± 5.8	33.5 ± 5.7	<0.001
Height (cm)	172.9 ± 5.9	160.2 ± 5.4	<0.001
Weight (kg)	74.2 ± 12.8	54.8 ± 10.2	<0.001
BMI (kg/m^2^)	24.8 ± 3.9	21.3 ± 3.7	<0.001
WC (cm)	84.1 ± 9.6	69.8 ± 8.4	<0.001
MET-hr (kcal/kg)	6.6 ± 14.6	4.2 ± 10.6	<0.001
Cumulative smoking exposure (pack-years)	31.4 ± 19.9	20.8 ± 17.7	<0.001
FPG (mmol/L)	100.8 ± 16.1	95.0 ± 10.6	<0.001
SBP (mmHg)	117.7 ± 13.9	102.8 ± 12.0	<0.001
DBP (mmHg)	74.8 ± 10.2	66.8 ± 9.0	<0.001
TC (mg/dL)	197.1 ± 37.3	185.2 ± 31.6	<0.001
HDL-C (mg/dL)	51.3 ± 11.3	65.8 ± 15.5	<0.001
LDL-C (mg/dL)	124.4 ± 34.4	104.6 ± 29.7	<0.001
TG (mg/dL)	149.2 ± 131.8	84.2 ± 54.7	<0.001
MetS			<0.001
Yes	1087 (12.1)	90 (3.8)	
No	7917 (87.9)	2264 (96.2)	

**Table 3:** Simple correlations between cumulative smoking exposure, demographic characteristics, MET-hr and biochemistry among all current smokers.

		Age	BMI	WC	MET-hr	FPG	SBP	DBP	TC	HDL-C	LDL-C	TG
All current smokers	Men	0.250	0.037	0.072	−0.163	0.078	0.005	0.045	0.080	−0.119	0.071	0.189
	Women	0.248	0.018	0.050	−0.087	0.048	0.001	0.030	0.106	−0.064	0.138	0.146
Current smokers under the age of 45	Men	0.237	0.052	0.077	−0.187	0.061	−0.001	0.034	0.090	−0.133	0.084	0.204
	Women	0.192	−0.018	0.006	−0.097	0.004	−0.038	0.005	0.045	−0.053	0.086	0.104

**Table 4:** Multiple logistic regression for predicting MetS in terms of age, sex, BMI, cumulative smoking exposure, and MET-hr among all current smokers.

	All Current Smokers			Current Smokers Under the Age of 45		
	Exp (B)	95%CI	*p*	Exp (B)	95%CI	*p*
Age	1.051	1.046–1.056	<0.001	1.062	1.053–1.071	<0.001
Sex (female vs. male)	0.524	0.470–0.583	<0.001	0.505	0.448–0.569	<0.001
BMI	1.264	1.247–1.281	<0.001	1.257	1.239–1.276	<0.001
Cumulative smoking exposure	1.004	1.002–1.006	<0.001	1.004	1.001–1.006	0.002
MET-hr	0.995	0.992–0.998	0.012	0.995	0.992–0.998	0.003

Adjusted for age, sex, body mass index (BMI), cumulative smoking exposure, and physical activity (MET-hr).

## Data Availability

Restrictions apply to the availability of these data. Data were obtained from the MJ Health Research Foundation and are available at 20 October 2025 [http://www.mjhrf.org/en/index.php?action=download&tid=1] with the permission of the MJ Health Research Foundation.

## Supplementary Materials

The following supporting information can be downloaded at: https://www.mdpi.com/article/10.3390/healthcare13212678/s1, Table S1: The calculation of cumulative smoking exposure; Table S2: Multiple logistic regression for predicting elevated WC in terms of age, sex, BMI, cumulative smoking exposure, and MET-hr among all current smokers; Table S3: Multiple logistic regression for predicting elevated TG in terms of age, sex, BMI, cumulative smoking exposure, and MET-hr among all current smokers; Table S4: Multiple logistic regression for predicting low HDL-C in terms of age, sex, BMI, cumulative smoking exposure, and MET-hr among all current smokers; Table S5: Multiple logistic regression for predicting elevated BP in terms of age, sex, BMI, cumulative smoking exposure, and MET-hr among all current smokers; Table S6: Multiple logistic regression for predicting elevated FPG in terms of age, sex, BMI, cumulative smoking exposure, and MET-hr among all current smokers.
