# Supplementary material for: Effects of Sex, Smoking, and Physical Activity on Metabolic Syndrome Among Current Smokers: A Cross-Sectional Study from Taiwan
Source: Healthcare (Basel). 2025 Oct 23;13(21):2678. doi: 10.3390/healthcare13212678 (PMC12610844; doi:10.3390/healthcare13212678)
Supplement: Supplementary file 1 [file healthcare-13-02678-s001.zip › healthcare-3847568-supplementary.pdf]

Supplementary Table S1. The calculation of cumulative smoking exposure

How many years have you been smoking (ex-smokers, answer based on past situations)? →duration	(1) Less than one year →1<br>(2) One to three years →2<br>(3) Three years to five years →3<br>(4) Five years to ten years →4<br>(5) More than ten years→5
Do you smoke? → frequency	(1) Don't smoke (those who choose not to smoke skip to the next big question) →0<br>(2) Don't smoke, but often smoke secondhand smoke →0<br>(3) Used to smoke, but now quit →1<br>(4) Smoke occasionally →2<br>(5) Smoke every day→3
How many cigarettes do you smoke per day on average? (Ex-smokers answer based on past situations) → amount	(1) Within five pieces →1<br>(2) Five to ten pieces (half pack) →2<br>(3) Half pack to one pack →3<br>(4) More than one packv→4

Supplementary Table S2. Multiple logistic regression for predicting elevated WC in terms of age, sex, BMI, cumulative smoking exposure, and MET-hr among all current smokers

	All current smokers			Current smokers under the age of 45		
	Exp (B)	95%CI	P	Exp (B)	95%CI	P
Age	1.037	1.021–1.053	<0.001	1.044	1.016–1.072	0.002
Sex (female vs. male)	10.402	7.020–15.415	<0.001	10.416	6.583–16.482	<0.001
BMI	2.417	2.271–2.573	<0.001	2.427	2.255–2.612	<0.001
Cumulative smoking exposure	1.002	0.996–1.009	0.488	0.998	0.990–1.006	0.626
MET-hr	0.990	0.980–1.000	0.041	0.995	0.983–1.006	0.358

elevated WC: men ≥102 cm, women ≥88 cm

Supplementary Table S3. Multiple logistic regression for predicting elevated TG in terms of age, sex, BMI, cumulative smoking exposure, and MET-hr among all current smokers

	All current smokers			Current smokers under the age of 45		
	Exp (B)	95%CI	P	Exp (B)	95%CI	P
Age	1.027	1.023–1.031	<0.001	1.082	1.072–1.092	<0.001
Sex (female vs. male)	0.377	0.325–0.438	<0.001	0.304	0.253–0.366	<0.001
BMI	1.216	1.202–1.230	<0.001	1.220	1.204–1.237	<0.001
Cumulative smoking exposure	1.013	1.011–1.015	<0.001	1.013	1.011–1.016	<0.001
MET-hr	0.993	0.990–0.996	<0.001	0.994	0.991–0.998	0.003

elevated TG: ≥150 mg/dL

Supplementary Table S4. Multiple logistic regression for predicting low HDL-C in terms of age, sex, BMI, cumulative smoking exposure, and MET-hr among all current smokers

	All current smokers			Current smokers under the age of 45		
	Exp (B)	95%CI	P	Exp (B)	95%CI	P
Age	1.003	0.997–1.008	0.338	1.004	0.993–1.015	0.485
Sex (female vs. male)	2.137	1.858–2.458	<0.001	2.265	1.931–2.656	<0.001
BMI	1.149	1.134–1.164	<0.001	1.151	1.135–1.168	<0.001
Cumulative smoking exposure	1.008	1.005–1.010	<0.001	1.009	1.006–1.012	<0.001
MET-hr	0.993	0.988–0.997	0.002	0.995	0.990–1.000	0.051

low HDL-C: <40 mg/dL in men; <50 mg/dL in women

Supplementary Table S5. Multiple logistic regression for predicting elevated BP in terms of age, sex, BMI, cumulative smoking exposure, and MET-hr among all current smokers

	All current smokers			Current smokers under the age of 45		
	Exp (B)	95%CI	P	Exp (B)	95%CI	P
Age	1.036	1.031–1.040	<0.001	1.037	1.027–1.046	<0.001
Sex (female vs. male)	0.266	0.222–0.319	<0.001	0.220	0.174–0.277	<0.001
BMI	1.167	1.153–1.180	<0.001	1.178	1.163–1.194	<0.001
Cumulative smoking exposure	0.999	0.997–1.001	0.534	0.999	0.996–1.002	0.460
MET-hr	1.002	0.999–1.005	0.120	1.001	0.997–1.004	0.621

elevated BP: SBP ≥130 mmHg; DBP, ≥85 mmHg

Supplementary Table S6. Multiple logistic regression for predicting elevated FPG in terms of age, sex, BMI, cumulative smoking exposure, and MET-hr among all current smokers

	All current smokers			Current smokers under the age of 45		
	Exp (B)	95%CI	P	Exp (B)	95%CI	P
Age	1.049	1.045–1.053	<0.001	1.063	1.055–1.071	<0.001
Sex (female vs. male)	0.489	0.438–0.545	<0.001	0.417	0.417–0.535	<0.001
BMI	1.127	1.116–1.139	<0.001	1.119	1.106–1.132	<0.001
Cumulative smoking exposure	1.000	0.998–1.002	0.900	1.000	0.998–1.002	0.862
MET-hr	0.996	0.993–0.999	0.002	0.996	0.993–0.999	0.021

elevated FPG: ≥100 mg/dL
